# Nutritional Enrichment and Sensory Profiles of Whole and Sifted Maize Flours Fortified With Jackfruit Seed Powder for Stiff Porridge Preparation

**DOI:** 10.1002/fsn3.72478

**Published:** 2026-10-20

**Authors:** Felix Ochieng Odhoch, Redemtor A. Ojwang, Mercy W. Mwaniki, Betty N. Mbatia, Mukiri E. Gitiri, Edward K. Muge, Evans N. Nyaboga

**Affiliations:** ^1^ Department of Biochemistry University of Nairobi Nairobi Kenya; ^2^ Department of Medical Biochemistry Maseno University Kisumu Kenya; ^3^ Department of Food Science and Technology Technical University of Kenya Nairobi Kenya; ^4^ United States International University–Africa Nairobi Kenya; ^5^ Kenya Bureau of Standards Nairobi Kenya

**Keywords:** functional food ingredient, mineral enrichment, nutrient‐dense stiff maize porridge, nutritional composition, underutilized resource

## Abstract

Jackfruit seeds are a rich source of protein, minerals and vitamins, making them a valuable underutilized by‐product that can be exploited for food enrichment to alleviate malnutrition. This study evaluated the effect of jackfruit seed powder on the nutritional profile and sensory attributes of white maize‐based stiff porridge. White maize seeds were milled into sifted (SMF) and whole maize flour (WMF), and separately blended with jackfruit seed powder (JKF) at different proportions ranging from 10% to 40%. Stiff maize porridge was prepared from each of the maize flour and maize flour blended with different proportions of JKF. The protein content of stiff porridge prepared from composite flours significantly increased from 1.66% to 5.95% and 1.24% to 5.98%, for SMF and WMF, respectively. There was a significant increase (*p* < 0.05) in vitamins B1 from 17.73 to 33.96 mg/kg and 14.88 to 33.99 mg/kg, for SMF and WMF, respectively, and B6 content from 3.72 to 17.14 mg/kg and 2.28 and 16.11 mg/kg, for SMF and WMF, respectively, in stiff porridge prepared from composite flours compared with maize flours. The analysis of mineral content showed a significant (*p* < 0.05) increase in calcium, copper, iron, potassium, magnesium and manganese levels with increase in the proportion of JKF in composite flours and their corresponding stiff porridge. The findings from sensory analysis showed that stiff porridge prepared from maize flours had higher acceptance compared to stiff porridge from composite flours. The incorporation of 10% JKF demonstrated an optimal balance between improved nutritional properties and sensory acceptability.

## Graphical abstract

The fortification of white maize flour with jackfruit seed powder significantly enhanced the nutritional value of stiff maize porridge. The increase in the proportion of jackfruit seed powder in maize‐jackfruit blended flours resulted in increased nutritional quality in the corresponding stiff maize porridge. These findings highlight the potential of jackfruit seed powder as a promising functional ingredient in the development of composite flour, offering nutritional benefits while maintaining acceptable sensory attributes.

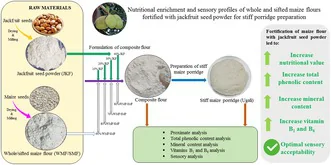

## 1 Introduction

According to Yilmaz and Yilmaz (2025) micronutrient deficiency affects over two billion people globally, mainly caused by inadequate intake of essential vitamins and minerals leading to stunted growth, delays in physical and cognitive development. Particularly among susceptible populations such as children, expectant mothers, and economically disadvantaged communities (Kemigisha 2025). Although significant strides have been made in combating global hunger, hidden hunger continues to pose a significant public health problem, presenting a need for sustainable, scalable and cost‐effective food‐based solutions, especially food fortification, to enhance micronutrient intake required for optimal health and development (Chaudhary et al. 2025).

White maize is a primary food source for many individuals in Sub‐Saharan Africa, particularly in Kenya (Khamila et al. 2020; Awata et al. 2019). White maize‐based stiff porridge (commonly referred to as *ugali* in East Africa), despite being a major dietary staple, provides limited quantities of essential amino acids such as lysine and tryptophan and is also low in B‐complex vitamins (Prasanna et al. 2001). Additionally, it has limited bioavailability of microminerals such as iron and zinc, as well as the absence of provitamin A (Deshmukh et al. 2023). Therefore, over‐reliance on maize‐based stiff porridge without dietary diversification increases the risk of nutritional deficiencies, especially in communities that rely heavily on it as their primary food source (Ekpa et al. 2019). There is therefore a need to fortify maize with locally available, nutrient‐rich and underutilized by‐products from crops such as seeds from jackfruit ( *Artocarpus heterophyllus* ).

Jackfruit is the largest fruit that weighs up to 50 kg among the edible fruits in the Moraceae family (Akter and Haque 2019). The fruit is composed of three main parts namely the pulp, seeds and the rind, which accounts for 30%–32%, 18% and 5%–55%, respectively. The rinds, rags, latex and seeds makes up 65% of the fruit and are inedible without processing (Ranasinghe et al. 2019). Ripe fruits are often eaten, but they can also be processed to make snacks and canned food products. Jackfruit seeds are often discarded as waste and yet the seeds possess untapped nutritional potential (Adan et al. 2020; Brahma and Ray 2023). Jackfruit seeds are an excellent source of protein, vitamins, dietary fiber and minerals (Swami et al. 2012). The seeds also contain bioactive compounds which have several health benefits like antimicrobial activities, anti‐aging, antioxidant and anti‐cancer. The seeds are edible when roasted, fried or cooked and its flour can be utilized to produce a variety of baked items such as cakes and bread (Noor et al. 2014; Ranasinghe et al. 2019). Overall, seeds of jackfruit are nutritious and underutilized by‐product with the potential for improving nutritional profile of white maize‐based foods to curb food and nutrition insecurity.

One of the main strategies to tackle malnutrition is through the fortification of foods with by‐products that are rich in micronutrients and vitamins. The incorporation of jackfruit seed powder in maize flour holds promise for enhancing the nutritional quality of stiff maize porridge. Jackfruit seeds are considered waste and have been reported to lack significant flavor (Mohamad et al. 2019). While jackfruit seed powder contributes valuable nutrients and bioactive compounds, its incorporation in stiff maize porridge may lead to undesirable changes in sensory attributes such as taste, aroma, and color, potentially reducing consumer acceptability. However, the use of jackfruit seed powder in the formulation of white maize flour used in stiff maize porridge has not been systematically studied. Consequently, there is limited information on the interactive effects of jackfruit seed powder and white maize flour on the nutritional value and sensory attributes of stiff maize porridge, and this represents a significant gap in functional food development.

This study sought to bridge the identified gap by evaluating the potential of jackfruit seed powder on the nutritional and sensory attributes of stiff maize porridge prepared from white maize flour supplemented with different proportions of jackfruit seed powder. Specifically, the study elucidated the synergistic effects of white maize flour and jackfruit seed powder thereby identifying the optimal blends that maximize nutritional fortification and sensory attributes. The study aimed to: (a) characterize the dose‐dependent impacts of jackfruit seed powder on the nutritional value of whole and sifted stiff maize porridge, and (b) establish optimal incorporation levels, balancing nutritional enhancement with sensory acceptability. We hypothesized that the incorporated jackfruit seed powder could significantly enhance the nutritional properties of maize stiff porridge, while sensory acceptability would depend on the overall balance of the formulation.

## 2 Materials and Methods

### 2.1 Source of Experimental Materials

Six mature and ripe jackfruits, each weighing approximately 20 kg, were purchased from a farmer in Kwale County, Kenya (4° 10′ 27.9984″ S and 39° 27′ 37.0008″ E). All the fruits were obtained from the same jackfruit tree. White maize grains (10 kg) were sourced from a farm (−0° 37′ 19.436″ N and 34° 19′ 51.731 E) in Homa Bay County, Kenya. All the materials were transported to the laboratory and stored at room temperature (23°C ± 2°C) for further processing.

### 2.2 Preprocessing of Jackfruit Seeds, Maize Grain, and Formulation of Composite Flour

The preparation of jackfruit seeds and maize grains flour was conducted as outlined by Arefin et al. (2022). The jackfruits were sliced open, seeds obtained, and peeled to remove the outer coating. The seeds were soaked in sterile distilled water for 24 h to remove the brown covering on the cotyledons. The seeds were then sliced and sun dried for 4 days before grinding into powder using a milling machine (model PRH‐2000A, Ho Chi Minh city, Vietnam). Seed powder was stored in airtight containers at 23°C ± 2°C until use.

Clean white maize grains were sundried and then packed into two separate batches. The two batches were processed into whole and sifted maize flour, respectively, using a milling machine at Kabansora Millers, Nairobi. For each category of maize flour (whole maize and sifted maize), five sets of blended composite flour samples were formulated by addition of jackfruit seed powder in different proportions, ranging from 0% to –40% (Table 1).

**TABLE 1:** Composition of the different formulated maize flour blended with jackfruit seed powder at different proportions.

Type of maize	Ingredient(s)	Control	Composite flour type			
Sifted maize		SMF	SMF1	SMF2	SMF3	SMF4
	Sifted maize flour (SMF) (%)	100	90	80	70	60
	Jackfruit seed powder (JKF) (%)	0	10	20	30	40
Whole maize		WMF	WMF1	WMF2	WMF3	WMF4
	Whole maize flour (WMF) (%)	100	90	80	70	60
	Jackfruit seed powder (JKF) (%)	0	10	20	30	40

*Note:* SMF1, SMF2, SMF3, and SMF4 represent blended composite flour containing 10%, 20%, 30%, and 40% jackfruit seed powder, respectively. SMF—Sifted maize flour, SMF1–90:10 (90% SMF and 10% JKF), SMF2–80:20 (80% SMF and 20% JKF), SMF3–70:30 (70% SMF and 30% JKF), and SMF4–60:40 (60% SMF and 40% JKF). WMF1, WMF2, WMF3, and WMF4 represent composite flours containing 10%, 20%, 30%, and 40% jackfruit seed powder, respectively. WMF—whole maize flour, WMF1–90:10 (90% WMF and 10% JKF), WMF2–80:20 (80% WMF and 20% JKF), WMF3–70:30 (70% WMF and 30% JKF), and WMF4–60:40 (60% WMF and 40% JKF). Each of the composite maize flour blended with jackfruit seed powder was prepared in triplicate.

### 2.3 Preparation of Stiff Maize Porridge (*Ugali*)

Approximately 100 g of each composite and control flour samples was progressively added to 200 mL of boiling water while stirring continuously over medium heat with a wooden stick for approximately 7 min. The stiff porridge was subsequently turned and allowed to cook on low heat for an additional 3 min. The prepared stiff maize porridge was used for nutritional, elemental and sensory analysis.

### 2.4 Nutritional Analysis of the Composite Flour and Stiff Maize Porridge

#### 2.4.1 Determination of Ash Content

The ash content was measured following the Association of Official Analytical Chemists (AOAC) method (AOAC 2000). About 5 g of each sample was measured into pre‐conditioned porcelain crucibles. Initially, the sample was carbonized over a flame to remove carbon residues, and then transferred in a muffle furnace at 550°C for 12 h. After ashing, the crucibles were cooled, and the final weights were recorded. Analysis was conducted in triplicate. The ash content (%) was computed as follows:

$$\mathrm{Ash}\;\text{content}\;\left(\%\right)=\frac{\text{Weight of}\;\mathrm{Ash}}{\text{Intiall sample weight}}\times 100.$$

#### 2.4.2 Determination of Moisture of Content

This was measured by the oven‐drying method according to AOAC (2000). Approximately 5 g of each sample was accurately weighed into moisture dishes, uniformly spread, and dried in an oven at 105°C for 17 h. After drying, the samples were weighed again, and the difference in weight was recorded. Each experimental analysis was conducted in triplicate. The moisture content was determined using the equation:

$$\text{Moisture content}\;\left(\%\right)=\frac{\left(W1-W2\right)}{\left(W1-W\right)}\times 100$$

where: mass of the empty dish (W), mass of the dish and sample before drying (W1), and mass of dish and sample after drying (W2).

#### 2.4.3 Determination of Crude Fat Content

The fat composition was measured as described in AOAC (2000) with minor modifications. 2 g of the processed sample was transferred into thimble, added 100 mL of petroleum ether to the extraction flask. The Soxhlet apparatus was assembled, and the process of extraction was performed under reflux for 2 h. Afterward, the solvent was evaporated, and the weight of the extracted fat was recorded. Each experimental analysis was conducted in triplicate. Fat content was computed as:

$$\mathrm{Fat}\;\text{content}\;\left(\%\right)=\frac{\text{Weight of extracted}\;\mathrm{fat}\;\left(g\right)}{\text{Weight of sample}\;\left(g\right)}\times 100$$

#### 2.4.4 Crude Protein Content Quantification

This was carried out using Kjeldahl digestion system (Model K‐370, Büchi Labortechnik AG, Germany) as outlined by AOAC (2000). Briefly 0.3 g of the sample was placed into a digestion tube, followed by the addition of a catalyst. Approximately 10 mL of concentrated sulfuric acid (H_2_SO_4_) was cautiously added to the digestion tube and thoroughly mixed. The tubes, along with the exhaust manifold, were then put in a preheated digester at 420°C. The heat shields were hooked onto the stand, and digestion was initiated with maximum airflow through the manifold for 3–5 min. The airflow was adjusted so that the fumes were just contained, and digestion was continued until the samples became clear, producing a green‐blue solution. After digestion, the samples were allowed to cool completely.

The distillation unit (Kjeldahl machine) was started and the batch setup began with a blank sample. The prepared digestion tube was positioned in place, and the safety door was securely closed. Once the distillation cycle was completed, the results were recorded and used for calculation of the crude protein content. The safety door was then opened, and the digestion tube was removed before proceeding with the next sample. Analysis of each sample was conducted in triplicate.

#### 2.4.5 Determination of Fiber Content

The crude fiber content was determined by acid‐Alkali hydrolysis (AOAC 2012) About 1.5 g of the defatted and dry sample was mixed with 100 mL of boiling sulfuric acid (H_2_SO_4_). A condenser was put on the flask and the mixture was boiled for 30 min with occasional swirling to provide even mixing. The sample was boiled and filtered and the filtered residue was transferred to another flask containing 100 mL of boiling NaOH solution. This boiling continued for another half hour with the same conditions. The residue was then filtered and washed with boiling water, hydrochloric acid (HCl) solution and petroleum ether to remove the remaining soluble ones. The sample was then placed in the oven at 105°C for 12 h, cooled and weighed. To measure the ash content, the dried residue was burnt in the muffle furnace at 550°C for 3 h. Each experimental analysis was conducted in triplicate. Crude fiber content was determined by difference between dried residue and ash.

#### 2.4.6 Determination of Carbohydrate Content

The available carbohydrate was calculated by subtracting the sum of moisture, ash, crude protein, crude fat, and crude fiber from 100%, as outlined by Zaria et al. (2012).

Available carbohydrate = 100 – [moisture (%) + crude protein (%) + crude fat (%) + ash (%) + crude fiber (%)].

### 2.5 Determination of Total Phenolic Content (TPC)

This was carried out according to the Folin–Ciocalteu method as documented by Kabubii et al. (2023). A 70% methanol solution was utilized to extract phenolic compounds from the samples. A 100 μL aliquot of the extract was mixed with 2.0 mL of 2% sodium carbonate (Na_2_CO_3_) solution, and after 2 min, 50 μL of 10% Folin–Ciocalteu reagent was added. The mixture was kept in the dark and incubated at 37°C for 30 min. Each experimental analysis was conducted in triplicate. The absorbance of the samples and the gallic acid standards was measured at 750 nm using a spectrophotometer. The total phenolic content was subsequently calculated from a calibration curve prepared with the gallic acid standards.

### 2.6 Mineral Content Analysis

#### 2.6.1 Inductively Coupled Plasma Optical Emission Spectroscopy

The content of mineral elements iron (Fe), calcium (Ca), magnesium (Mg), manganese (Mn) and copper (Cu) of the enriched flour samples was analyzed by Agilent 5110 Inductively Coupled Plasma Optical Emission Spectroscopy (ICP‐OES) model (Agilent technologies, Santa Clara California, USA) as outlined by Phan‐Thien et al. (2012). After ashing 3 g of the sample overnight in a muffle furnace, it was combined with 10 mL of nitric acid. The mixture was gently heated on a hot plate until a clear solution was achieved. The solution was subsequently diluted to 50 mL using distilled water. The mineral content was determined based on the characteristic emission spectra of each element using ICP‐OES. Each experimental analysis was conducted in triplicate.

#### 2.6.2 Flame Emission Spectrophotometer (FES)

Potassium (K) analysis was performed using 410 Corning Flame Emission Spectrophotometer (FES) model (Halstead, Essex, England) according to Adan et al. (2020). Prior to analysis, a wet digestion method was employed to solubilize the flour samples. Precisely 1 g of the sample was weighed into a beaker and digested using 60% nitric acid (HNO_3_). The beakers were then heated on a hot plate inside a fume hood for approximately 30 min. The digestion process yielded a clear solution, confirming the completion of mineralization. The digested samples were placed in 50 mL centrifuge tubes, and deionized distilled water was added to bring the solution to the mark. For the FES analysis, the instrument was calibrated and set to the appropriate wavelength of 765 nm, which is optimal for detecting potassium (K). Each experimental analysis was conducted in triplicate.

### 2.7 Vitamins B1 and B6 Quantification Using High‐Performance Liquid Chromatography

These vitamins were analyzed using Shimadzu prominence LC‐20 ad as outlined by Sarwar et al. (2020). The **s**amples were prepared by extraction of 2.5 g of the sample in 25 mL of the prepared extraction solution (made from a mixture of acetonitrile and glacial acetic acid, filtered through a 0.45 μm membrane filter). The extracted samples were subsequently incubated in a shaking water bath at 70°C for 40 min. The samples were then allowed to cool to room temperature and centrifuged at 3000 rpm for 10 min. The supernatant was filtered through a 0.45 μm membrane filter, 1 mL of the filtrate was transferred into an HPLC vial. The mobile phase was made up of ultra‐pure water with 1% acetonitrile and glacial acetic acid in gradient with a flow of 0.3 mL/min. The injection volume was 8 μL and the instrument was set to the appropriate wavelength of 234 and 266 nm, optimal for detection of B1 and B6, respectively. The standards were prepared using thiamine hydrochloride and pyridoxine hydrochloride dissolved in 25 mL of HPLC‐grade water, for quantification of vitamins B1 and B6, respectively. Each experimental analysis was conducted in triplicate.

### 2.8 Sensory Evaluation of Fortified Stiff Maize Porridge

Sensory assessment was done following the protocol outlined by Murage et al. (2021). A panel of 31 Food Science and Technology students (trained assessors) from the Department of Food Science and Technology at the Technical University of Kenya with knowledge and experience in sensory testing was recruited. All the stiff maize porridge sample groups were simultaneously served to the panelists in equal quantities and with a uniform presentation in white plastic dishes. Panelists received bottled mineral water to rinse their mouths between samples. A 5‐point hedonic scale ranging from 1 to 5 was utilized to assess the fortified stiff maize porridge samples. The scale was defined as follows: 1 indicated “dislike extremely,” 2 “dislike a little,” 3 “neutral response” (“neither like nor dislike”), 4 “like a little,” and 5 “like extremely.” Each sample was randomly assigned a four‐character code to eliminate bias. The stiff maize porridge samples were evaluated based on their aroma, color, texture, taste, appearance, mouthfeel, and overall acceptability.

### 2.9 Statistical Data Analysis

All evaluations of samples and controls were done in triplicate. All data were subjected to one‐way ANOVA to determine whether significant differences existed among the treatment means and Tukey's honestly significant difference (HSD) test was performed for post hoc treatment mean comparison at *p* < 0.05. Statistical analysis was conducted using RStudio (Version 2025.05.0 Build 496, “Mariposa Orchid” Release, Posit Software, PBC, Chicago, IL, USA) with the support of the agricolae package for multiple comparison procedures.

Graphs illustrating the sensory and nutritional characteristics of the composite flour blends were created using SigmaPlot for Windows, Version 15.0.0.13. The graphical representations, including group error bar plots, were generated to display the mean values along with standard deviations, enabling a clear visualization of variation among replicates.

## 3 Results

### 3.1 Proximate and Physicochemical Properties of Fortified Maize Flour and Stiff Maize Porridge

The ash levels of maize flour blended with different proportions of jackfruit seed powder (JKF) and the corresponding stiff maize porridge are represented in Figure 1. Jackfruit seed powder (JKF) recorded the highest ash content (3.82%), while sifted maize flour (SMF) had the lowest (0.70%). Upon blending SMF flour with 40% JKF, ash content showed a significant increase (*p* < 0.05) to 2.15% as compared to SMF. The ash content increased significantly (*p* < 0.05) in stiff maize porridge supplemented with 40% JKF (0.83%) as compared to SMF stiff porridge (0.33%). Similarly, upon blending WMF with JKF, the ash content significantly (*p* < 0.05) increased in stiff maize porridge supplemented with 40% JKF to 2.24% compared with WMF stiff porridge. The ash content significantly (*p* < 0.05) increased in stiff maize porridge supplemented with 40% JKF (0.89%) as compared to WMF stiff maize porridge (0.49%). As the level of jackfruit seed flour increased in the composite SMF and WMF, the ash content increased significantly (Figure 1). The data showed a significant difference (*p* < 0.05) in ash content between the maize flour blends and their corresponding stiff maize porridge.

**FIGURE 1:**
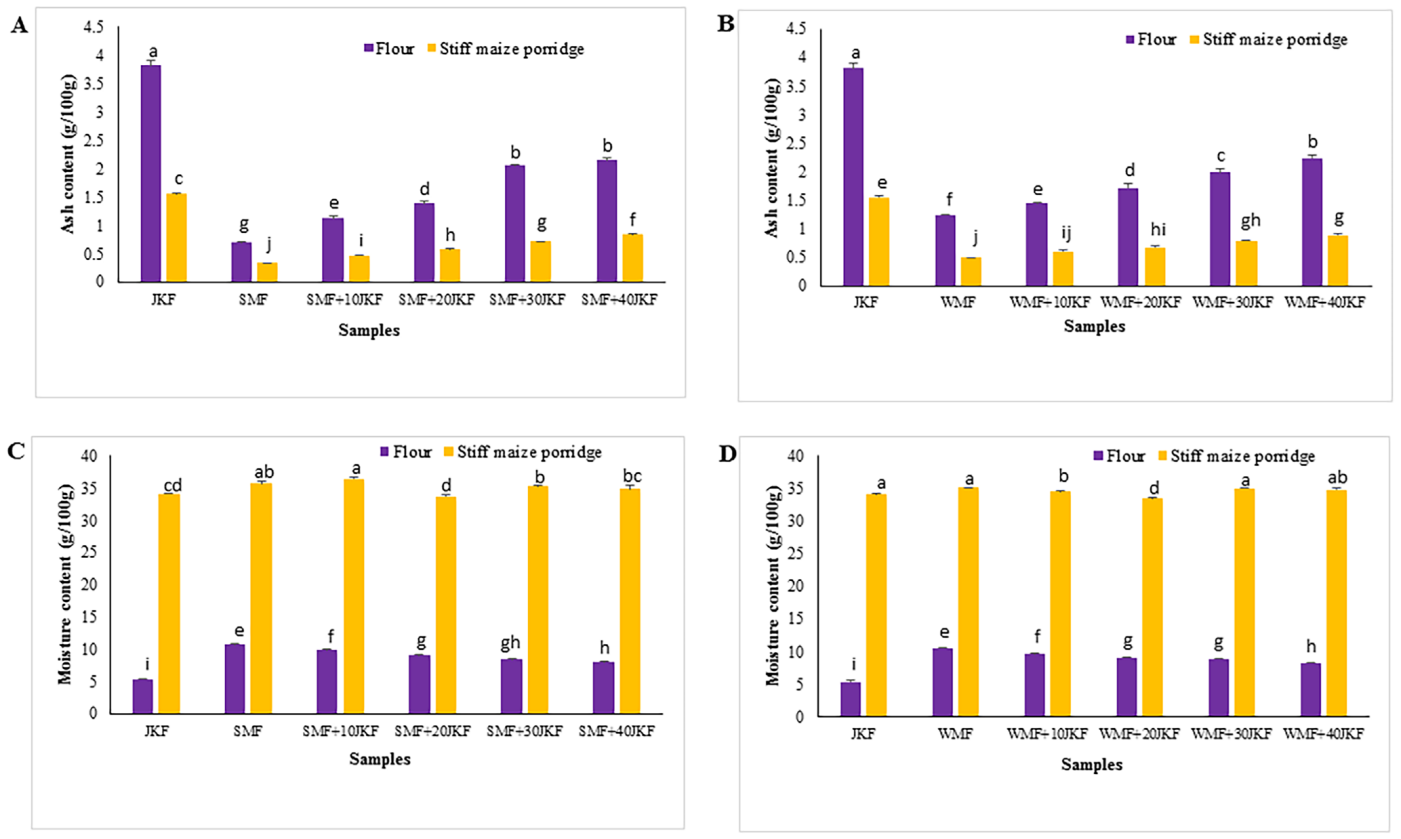
Ash and moisture content of jackfruit seed powder (JKF), sifted maize flour (SMF), whole maize flour (WMF) as well as SMF and WMF blended with different proportions of jackfruit seed powder and their corresponding stiff maize porridge. A and B represent ash content of SMF and WMF, respectively, blended with different proportions of JKF and their corresponding stiff maize porridge; C and D represent moisture content of SMF and WMF, respectively, blended with different proportions of JKF and their corresponding stiff maize porridge. SMF + 10JKF (90% SMF and 10% JKF), SMF + 20JKF (80% SMF and 20% JKF), SMF + 30JKF (70% SMF and 30% JKF), SMF + 40JKF (60% SMF and 40% JKF), WMF + 10JKF (90% WMF and 10% JKF), WMF + 20JKF (80% WMF and 20% JKF), WMF + 30JKF (70% WMF and 30% JKF), and WMF + 40JKF (60% WMF and 40% JKF). Values represent mean values ± standard deviation (*n* = 3). Significant differences (*p* ≤ 0.05) are denoted by different letters as determined by Tukey's honestly significant difference (HSD) test.

The moisture content of maize flour blended with different proportions of jackfruit seed flour and their corresponding stiff maize porridge is presented in Figure 1. The findings showed that jackfruit seed flour (JKF) had the lowest moisture content (5.35%), whereas sifted maize flour (SMF) recorded the highest (10.72%). When SMF was blended with 40% JKF, the moisture content significantly (*p* < 0.05) decreased to 8.00%. A steady decline in moisture was observed with increasing proportions of JKF, although no significant difference (*p* > 0.05) occurred between SMF + 20% JKF, SMF + 30% JKF and SMF + 40% JKF. Whole maize flour (WMF) had the highest (10.53%) moisture content recorded for the maize flour. Upon blending WMF with 40% JKF, the moisture content significantly (*p* < 0.05) reduced to 8.19%.

Moisture content of stiff maize porridge was significantly (*p* < 0.05) higher compared to the composite flours. SMF stiff porridge had 35.64% moisture content, while stiff maize porridge from SMF blended with 40% JKF recorded 34.86%. There was no statistical significance (*p* > 0.05) observed between most maize flour blended with jackfruit seed powder for both SMF and WMF (Figure 1). Stiff porridge from WMF blended with 40% JKF (34.86%) showed significantly (*p* < 0.05) higher moisture content compared with WMF stiff porridge (34.10%).

Protein content of the maize flour blended with different proportions of JKF and their corresponding stiff maize porridge is presented in Figure 2. Protein content differed significantly (*p* < 0.05) across the different formulations of flour and stiff maize porridge. The protein content in SMF, WMF, and JKF was 8.03%, 9.13%, and 15.49%, respectively. There was a significant (*p* < 0.05) increase in protein content in stiff maize porridge prepared from maize flour blended with different proportions of JKF compared to stiff maize porridge prepared from either SMF or WMF only. For SMF, the protein content of stiff porridge prepared from SMF‐jackfruit blended flour significantly increased from 1.66% to 5.95%, while for stiff maize porridge prepared from WMF‐jackfruit blended flour increased from 1.24% to 5.98%. The cooked jackfruit seed powder (100% JKF) sample exhibited a protein content of 7.23%, which is lower than the uncooked jackfruit seed powder (15.49%). The data revealed a significant difference (*p* < 0.05) between the flours and their stiff maize porridge counterparts.

**FIGURE 2:**
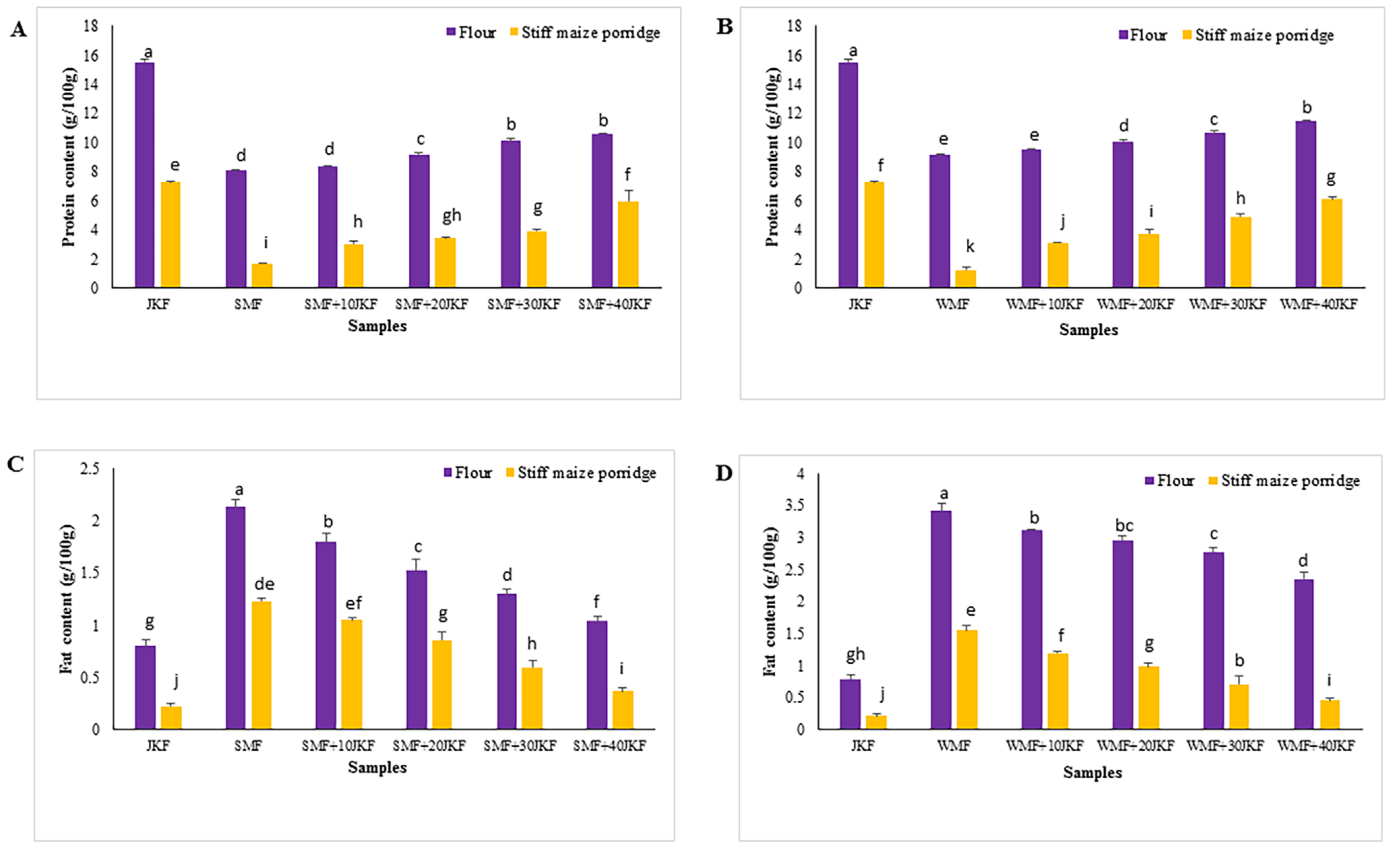
Protein and crude fat content (%) of jackfruit seed powder (JKF), sifted maize flour (SMF), whole maize flour (WMF) as well as SMF and WMF blended with different proportions of jackfruit seed powder and their corresponding stiff maize porridge. A and B represent protein content of SMF and WMF, respectively, blended with different proportions of JKF and their corresponding stiff maize porridge; C and D represent crude fat content of SMF and WMF, respectively, blended with different proportions of JKF and their corresponding stiff maize porridge. SMF + 10JKF (90% SMF and 10% JKF), SMF + 20JKF (80% SMF and 20% JKF), SMF + 30JKF (70% SMF and 30% JKF), SMF + 40JKF (60% SMF and 40% JKF), WMF + 10JKF (90% WMF and 10% JKF), WMF + 20JKF (80% WMF and 20% JKF), WMF + 30JKF (70% WMF and 30% JKF), and WMF + 40JKF (60% WMF and 40% JKF). The values represent mean values ± standard deviation (*n* = 3). Different lowercase letters in different bars indicate significant differences at 5% level. Significant differences (*p* ≤ 0.05) are denoted by different letters as determined by Tukey's honestly significant difference (HSD) test.

Fat content was analyzed in both composite flour blends and their corresponding stiff maize porridge. As presented in Figure 2, the fat quantity varied significantly (*p* < 0.05) across the different maize flour formulations. Sifted maize flour (SMF) had a fat content of 2.13%, which significantly (*p* < 0.05) decreased to 1.04% when blended with 40% jackfruit seed flour (SMF + 40JF). Stiff maize porridge prepared from SMF and SMF + 40JKF, fat content was significantly (*p* < 0.05) reduced to 1.23% for SMF and 0.36% for SMF + 40JKF. Similarly, whole maize flour (WMF) had the highest fat content among the three flours at 3.42%, which significantly (*p* < 0.05) reduced to 2.36% in the WMF + 40JF, and further decreased to 1.56% and 0.46% in their respective stiff maize porridge samples. Jackfruit seed powder (JKF) had a significantly (*p* < 0.05) low‐fat content of 0.80%, and its cooked form had the lowest at 0.22%.

The crude fiber content of maize flour blended with jackfruit seed powder and their corresponding stiff maize porridge is presented in Figure 3. The data showed that jackfruit seed flour (JKF) recorded the highest fiber level (3.40%), while sifted maize flour (SMF) had the lowest (2.33%). Blending SMF with JKF significantly (*p* < 0.05) increased the fiber content with SMF + 40% JKF recording the highest content (3.26%). The fiber content significantly (*p* < 0.05) increased in stiff maize porridge supplemented with 40% JKF (2.49%) compared to SMF stiff porridge (1.30%). Similarly, the fiber content of stiff maize porridge from WMF supplemented with 40% JKF (2.54%) was significantly higher than WMF stiff maize porridge (1.33%).

**FIGURE 3:**
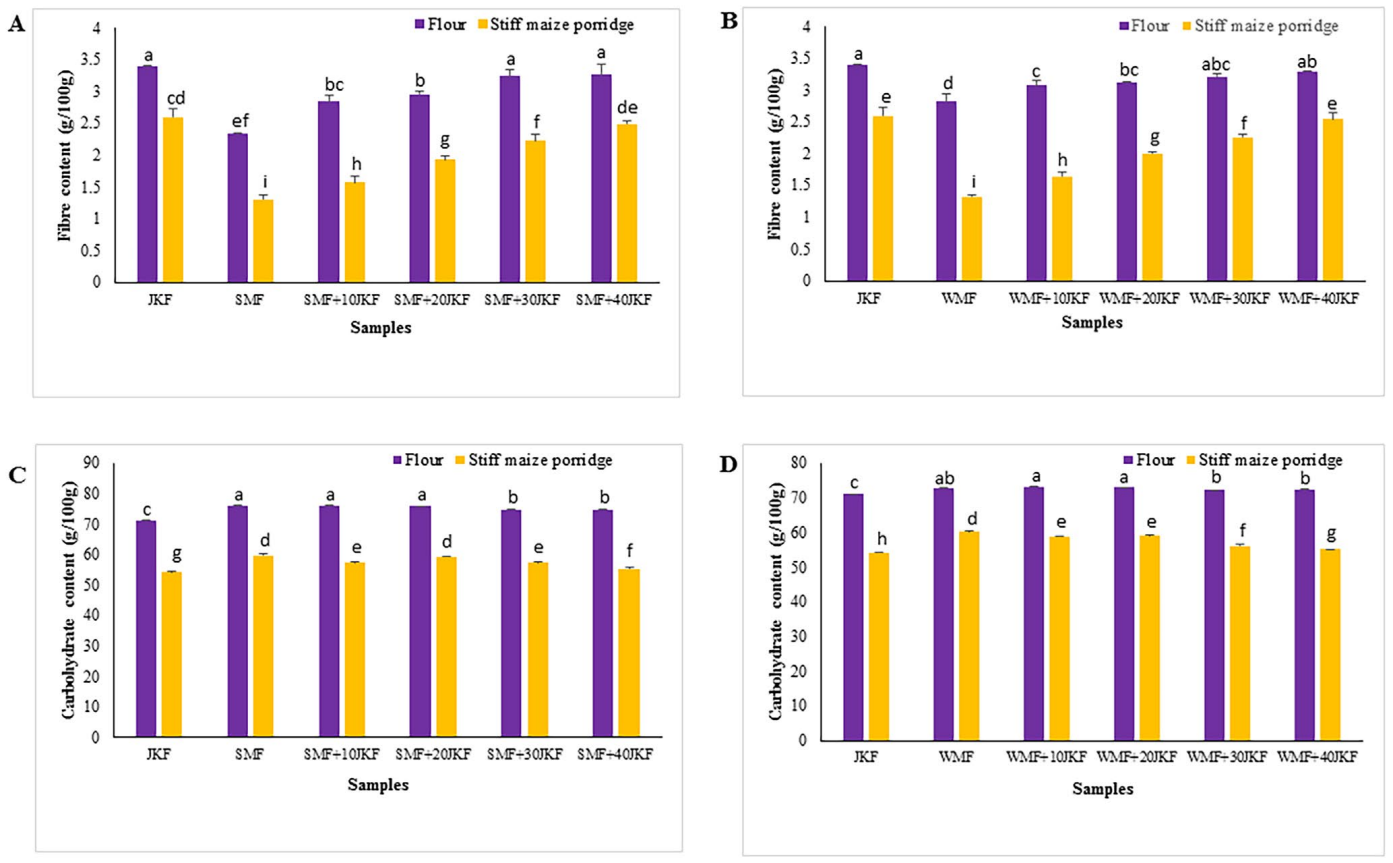
Fiber and carbohydrate content (g/100 g) of jackfruit seed powder (JKF), sifted maize flour (SMF), whole maize flour (WMF) as well as SMF and WMF blended with different proportions of jackfruit seed powder and their corresponding stiff maize porridge. A and B represent fibre content of SMF and WMF, respectively, blended with different proportions of JKF and their corresponding stiff maize porridge; C and D represent carbohydrate content of SMF and WMF, respectively, blended with different proportions of JKF and their corresponding stiff maize porridge. SMF + 10JKF (90% SMF and 10% JKF), SMF + 20JKF (80% SMF and 20% JKF), SMF + 30JKF (70% SMF and 30% JKF), SMF + 40JKF (60% SMF and 40% JKF), WMF + 10JKF (90% WMF and 10% JKF), WMF + 20JKF (80% WMF and 20% JKF), WMF + 30JKF (70% WMF and 30% JKF), and WMF + 40JKF (60% WMF and 40% JKF). The values represent mean values ± standard deviation (*n* = 3). Different lowercase letters in different bars indicate significant differences at the 5% level. Significant differences (*p* ≤ 0.05) are denoted by different letters as determined by Tukey's honestly significant difference (HSD) test.

Carbohydrate content of maize flour fortified with different proportions of jackfruit seed powder and their corresponding stiff maize porridge is presented in Figure 3. The carbohydrate content of jackfruit seed powder (71.14%) was significantly reduced (*p* < 0.05) compared to sifted maize flour (SMF) and whole maize flour (WMF) at 76.08% and 72.85%, respectively. Blending maize flour with jackfruit seed powder significantly reduced the total carbohydrate content; although in SMF + 30JKF and SMF + 40JKF formulations, the reduction was not significant.

### 3.2 Phenolic Content of Fortified Maize Flour and Stiff Maize Porridge

Phenolic content of maize flour blended with different proportions of jackfruit seed powder and their corresponding stiff maize porridge is presented in Figure 4. Jackfruit seed powder (JKF) recorded the highest phenolic content (2.22 mg/g) and differed significantly (*p* < 0.05) from SMF (0.57 mg/g) and WMF (1.13 mg/g). Sifted maize blended with JKF had a consistent increase in phenolic content for both the composite flour and stiff maize porridge. The increase in jackfruit seed powder proportion from 10% to 40% reflected a significant increase (*p* < 0.05) in phenolic contents in a progressive manner. Stiff maize porridge samples had significantly lower phenolic contents compared to their uncooked flour counterparts. Samples with higher jackfruit proportions retained significantly more phenolics (e.g., SMF + 40JKF stiff porridge: 1.95 mg/g; WMF + 40JKF stiff porridge: 2.16 mg/g) than their unfortified maize flour (SMF stiff porridge: 0.17 mg/g; WMF stiff porridge: 0.58 mg/g), ‘(*p* < 0.05)’. However, not all sample comparisons showed significant differences. For instance, in the WMF series, WMF + 30JKF and WMF + 40JKF showed no significant differences (*p* > 0.05) between them. Similarly, some stiff maize porridge samples such as WMF + 20JKF and WMF + 30JKF showed no significant differences.

**FIGURE 4:**
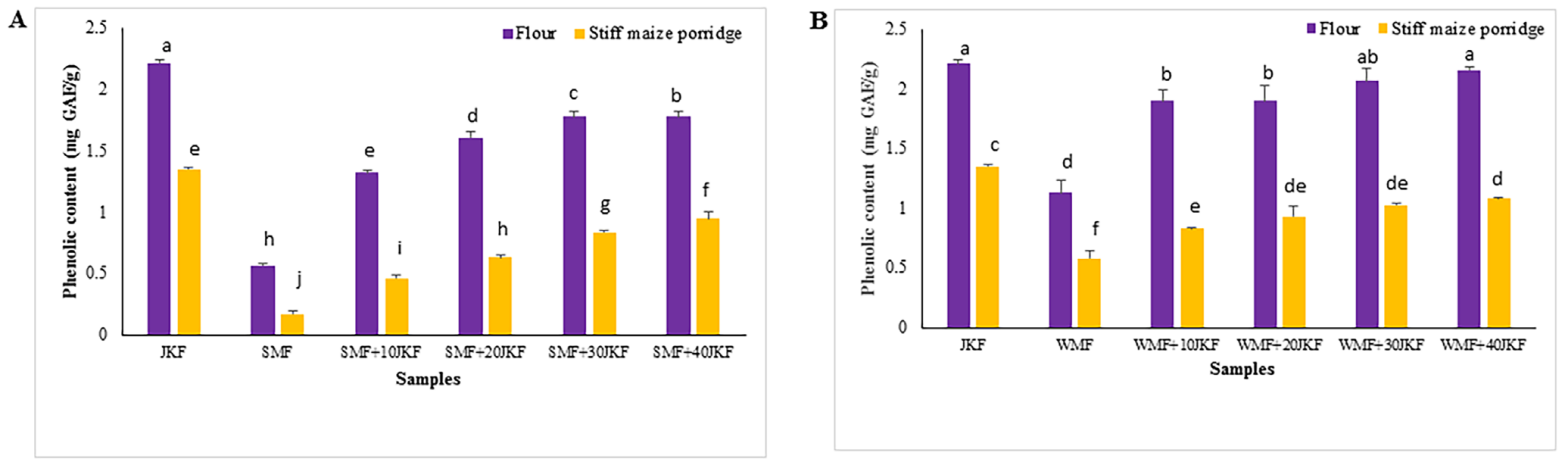
Total phenolic content (mg gallic acid equivalents (GAE)/g) of jackfruit seed powder (JKF), maize flour (SMF and WMF), composite flour (maize flour blended with different proportions of jackfruit seed powder) and their corresponding stiff maize porridge. A and B represent total phenolic content of SMF and WMF, respectively, blended with different proportions of JKF and their corresponding stiff maize porridge. SMF + 10JKF (90% SMF and 10% JKF), SMF + 20JKF (80% SMF and 20% JKF), SMF + 30JKF (70% SMF and 30% JKF), SMF + 40JKF (60% SMF and 40% JKF), WMF + 10JKF (90% WMF and 10% JKF), WMF + 20JKF (80% WMF and 20% JKF), WMF + 30JKF (70% WMF and 30% JKF), and WMF + 40JKF (60% WMF and 40% JKF). Values represent means ± standard deviations (*n* = 3). Different lowercase letters in different bars indicate significant differences at the 5% level. Significant differences (*p* ≤ 0.05) are denoted by different letters as determined by Tukey's honestly significant difference (HSD) test. JKF, jackfruit seed powder; SMF, sifted maize flour; WMF, whole maize flour.

### 3.3 Vitamin B1 and B6 Content of Fortified Maize Flour and Stiff Maize Porridge

A significant increase (*p* < 0.05) in thiamine (B1) content was observed with increasing proportions of jackfruit seed powder in both SMF and WMF (Figure 5). Thiamine was not detected in sifted maize flour (SMF) and whole maize flour (WMF), but was present at significantly (*p* < 0.05) higher levels in the fortified maize flour containing jackfruit seed powder. In SMF blended with different proportions of JKF, thiamine ranged from 42.77 mg/kg in SMF + 10JKF to 134.56 mg/kg in SMF + 40JKF, while in their corresponding stiff porridge, thiamine content significantly (*p* < 0.05) decreased. Similarly, WMF blended with different proportions of JKF had thiamine levels increased from 43.03 (WMF + 10JKF) to 119.17 mg/kg (WMF + 40JKF), while the stiff porridge samples thiamine content significantly (*p* < 0.05) decreased. Jackfruit seed powder had a high thiamine content of 311.56 mg/kg, which decreased to 109.33 mg/kg after preparation of stiff maize porridge. However, not all differences in vitamin B1 between the samples were significant (*p* > 0.05).

**FIGURE 5:**
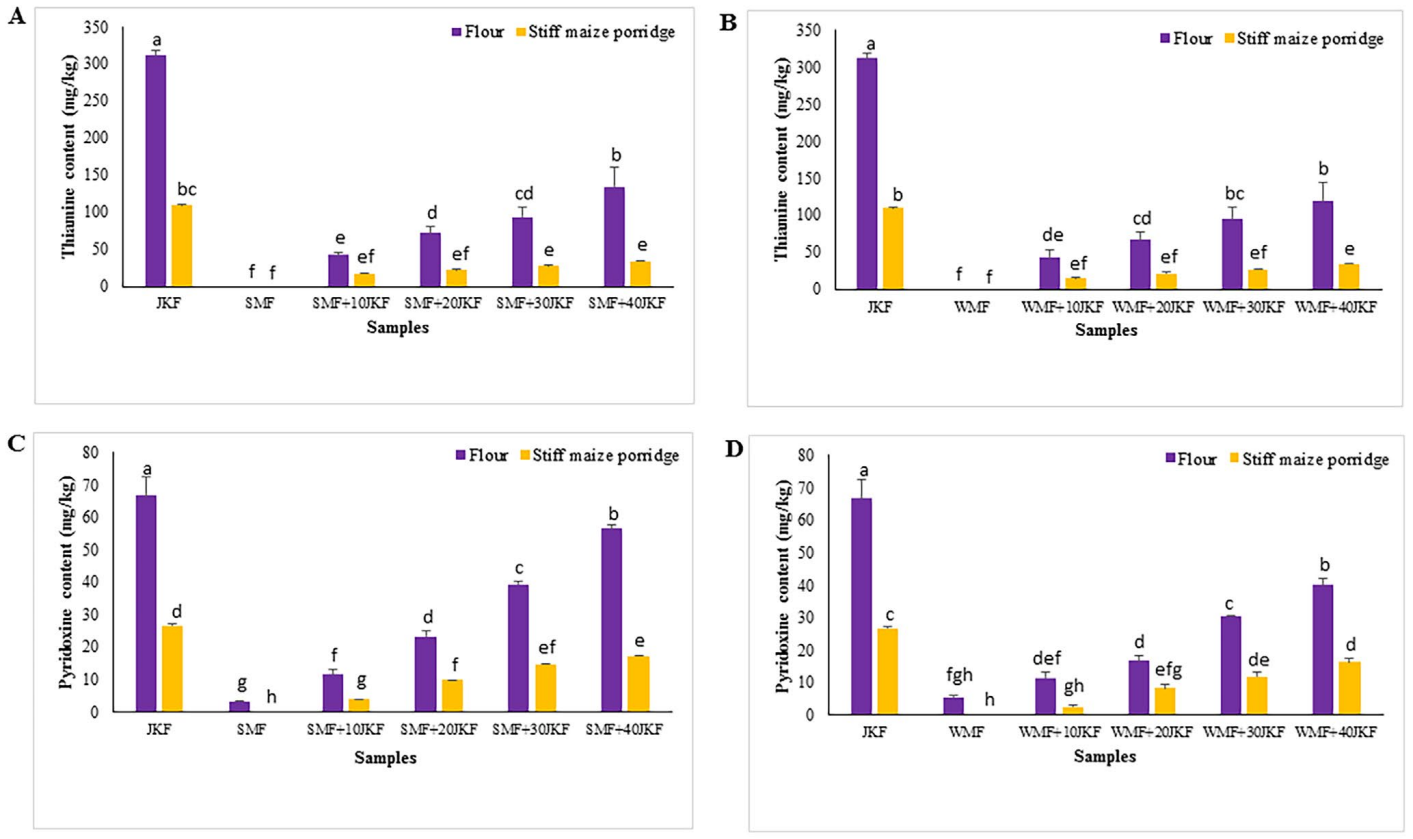
Thiamine (vitamin B1) and pyridoxine content (mg/kg) in jackfruit seed powder (JKF), sifted maize flour (SMF), whole maize flour (WMF), as well as SMF and WMF blended with different proportions of jackfruit seed powder and their corresponding stiff maize porridge. A and B represent thiamine (vitamin B1) content of SMF and WMF, respectively, blended with different proportions of JKF and their corresponding stiff maize porridge; C and D represent pyridoxine (vitamin B6) content of SMF and WMF, respectively, blended with different proportions of JKF and their corresponding stiff maize porridge. SMF + 10JKF (90% SMF and 10% JKF), SMF + 20JKF (80% SMF and 20% JKF), SMF + 30JKF (70% SMF and 30% JKF), SMF + 40JKF (60% SMF and 40% JKF), WMF + 10JKF (90% WMF and 10% JKF), WMF + 20JKF (80% WMF and 20% JKF), WMF + 30JKF (70% WMF and 30% JKF), and WMF + 40JKF (60% WMF and 40% JKF). The values represent mean values ± standard deviation (*n* = 3). Different lowercase letters in different bars indicate significant differences at the 5% level.

A significant increase (*p* < 0.05) in vitamin B6 content was observed as the proportion of jackfruit seed powder increased in both SMF and WMF (Figure 5). SMF and WMF had low B6 concentrations of 1.89 mg/kg and 1.98 mg/kg, respectively. With the blending of maize flour with different proportions of jackfruit seed powder, B6 significantly increased, ranging from 11.39 (SMF + 10JKF) to 56.32 mg/kg (SMF + 40JKF) for sifted maize and from 11.17 (WMF + 10JKF) to 39.94 mg/kg (WMF + 40JKF) for whole maize. However, preparation of stiff maize porridge significantly reduced vitamin B6 content (*p* < 0.05). In SMF stiff porridge, B6 was undetectable and ranged from 3.72 (SMF + 10JKF) to 17.14 mg/kg (SMF + 40JKF) in stiff porridge prepared from blended flour. Similarly, WMF stiff maize porridge had B6 levels between 2.28 (WMF + 10JKF) and 16.11 mg/kg (WMF + 40JKF), with no detectable B6 in whole maize stiff porridge. Jackfruit seed powder exhibited a high B6 content of 66.65 mg/kg, which declined significantly to 26.49 mg/kg after preparation of stiff porridge.

### 3.4 Mineral Content of Fortified Maize Flour and Stiff Maize Porridge

The mineral profile of maize flour and stiff maize porridge are presented in Tables 2 and 3. Generally, the analysis showed a significant (*p* < 0.05) increase in calcium, copper, iron, potassium, magnesium, and manganese content with an increase in jackfruit seed powder in SMF and WMF. However, not all maize flour blended with jackfruit seed powder showed a statistically significant change. From the findings, jackfruit seed powder recorded significantly higher mineral content (*p* < 0.05) compared to both sifted and whole maize flour blended with 40% jackfruit seed powder.

**TABLE 2:** Mineral composition (mg/100 g) of sifted maize flour fortified with different proportions of jackfruit seed powder and their corresponding stiff porridge.

Flour and stiff porridge samples	Calcium	Copper	Iron	Potassium	Magnesium	Manganese
JKF	110.68 ± 1.62^a^	1.06 ± 0.06^a^	5.15 ± 0.12^a^	34.91 ± 0.29^a^	132.10 ± 0.85^a^	1.1 ± 0.06^a^
SMF	12 ± 0.13^f^	0.12 ± 0.01^ef^	1.90 ± 0.12^d^	4.41 ± 0.44^hi^	51.33 ± 0.77^e^	0.32 ± 0.02^ef^
SMF + 10JKF	19.39 ± 0.99^e^	0.23 ± 0^de^	2.05 ± 0.07^d^	7.61 ± 0.50^g^	60.43 ± 0.73^d^	0.43 ± 0.01^de^
SMF + 20JKF	31.41 ± 1.94^d^	0.26 ± 0^de^	2.19 ± 0.08^cd^	10.48 ± 0.29^ef^	63.38 ± 0.70^d^	0.51 ± 0.01^cd^
SMF + 30JKF	39.88 ± 1.91^c^	0.33 ± 0.02^cd^	2.63 ± 0.01^c^	13.36 ± 1.15^d^	68.69 ± 0.83^g^	0.57 ± 0.02^c^
SMF + 40JKF	48.22 ± 0.67^b^	0.53 ± 0.06^b^	3.51 ± 0.01^b^	16.23 ± 0.50^c^	81.93 ± 0.64^b^	0.69 ± 0.04^b^
JKF*	47.38 ± 1.15^b^	0.45 ± 0.08^bc^	2.09 ± 0.17^d^	24.92 ± 0.72^b^	68.41 ± 1.14^c^	0.39 ± 0^e^
SMF*	3.56 ± 0.77^g^	0.03 ± 0.01^f^	0.57 ± 0^fg^	3.65 ± 0.23^i^	16.01 ± 0.52^i^	0.09 ± 0^i^
SMF + 10JKF*	6.92 ± 0.69^fg^	0.17 ± 0.01^def^	0.62 ± 0.01^fg^	6.69 ± 0.48^gh^	18.41 ± 0.41^i^	0.12 ± 0^hi^
SMF + 20JKF*	9.87 ± 1.20^d^	0.21 ± 0^de^	0.77 ± 0.04^fg^	9.09 ± 0^fg^	23.17 ± 0.46^h^	0.19 ± 0^ghi^
SMF + 30JKF*	18.84 ± 0.62^e^	0.23 ± 0.01^de^	1.04 ± 0.10^ef^	12.20 ± 0.48^de^	35.24 ± 0.46^g^	0.21 ± 0.01^fgh^
SMF + 40JKF*	20.97 ± 0.62^e^	0.34 ± 0.02^cd^	1.43 ± 0.13^e^	14.36 ± 0.63^cd^	40.07 ± 0.43^f^	0.26 ± 0.01^fg^
*p*	< 0.05	< 0.05	< 0.05	< 0.05	< 0.05	< 0.05

*Note:* SMF + 10JKF (90% SMF and 10% JKF), SMF + 20JKF (80% SMF and 20% JKF), SMF + 30JKF (70% SMF and 30% JKF), and SMF + 40JKF (60% SMF and 40% JKF). The values represent mean values ± standard deviation (*n* = 3). Different lowercase letters in same column indicate significant differences at 5% level.

Abbreviations: JKF, jackfruit seed powder; SMF, sifted maize flour.

* Represents stiff porridge from the corresponding flour.

**TABLE 3:** Mineral composition (mg/100 g) of whole maize flour fortified with different proportions of jackfruit seed powder and their corresponding stiff porridge.

Flour and stiff porridge samples	Calcium	Copper	Iron	Potassium	Magnesium	Manganese
JKF	110.68 ± 1.62^a^	1.06 ± 0.06^a^	5.15 ± 0.12^a^	34.91 ± 0.29^a^	132.10 ± 0.85^a^	1.1 ± 0.06^a^
WMF	13.98 ± 1.41^f^	0.13 ± 0.03^f^	2.01 ± 0.12^de^	5.49 ± 0.55^fg^	50.71 ± 0.68^f^	0.37 ± 0.01^ef^
WMF + 10JKF	22.36 ± 1.38^e^	0.24 ± 0.02^def^	2.25 ± 0.04^cd^	10.48 ± 0.57^e^	62.12 ± 0.31^e^	0.49 ± 0.02^de^
WMF + 20JKF	32.55 ± 0.66^d^	0.32 ± 0.01^cde^	2.53 ± 0.07^cd^	12.21 ± 0.57^de^	70.88 ± 0.19^d^	0.59 ± 0.01^cd^
WMF + 30JKF	42.27 ± 0.90^c^	0.37 ± 0.01^bcd^	2.76 ± 0.18^bc^	15.66 ± 0.29^c^	75.14 ± 0.51^c^	0.65 ± 0.01^bc^
WMF + 40JF	51.36 ± 0.47^b^	0.51 ± 0.02^b^	3.13 ± 0.20^b^	16.52 ± 0.29^c^	84.55 ± 0.10^b^	0.76 ± 0.05^b^
JKF*	47.38 ± 1.15^bc^	0.45 ± 0.08^bc^	2.09 ± 0.17^de^	24.92 ± 0.72^b^	68.41 ± 1.14^d^	0.39 ± 0^e^
WMF*	6.23 ± 0.433^g^	0.08 ± 0^f^	0.61 ± 0^g^	4.79 ± 0.88^g^	17.66 ± 0.33^i^	0.09 ± 0^h^
WMF + 10JKF*	8.34 ± 0.34^g^	0.18 ± 0.01^ef^	0.70 ± 0.01^g^	7.89 ± 0.24^f^	20.04 ± 0.58^i^	0.12 ± 0.01^h^
WMF + 20JKF*	11.01 ± 1.04^fg^	0.21 ± 0^def^	0.85 ± 0.02^g^	11.24 ± 0.42^e^	25.09 ± 0.57^h^	0.19 ± 0.01^gh^
WMF + 30JKF*	19.49 ± 1.03^e^	0.24 ± 0.01^def^	1.09 ± 0.06^fg^	11.96 ± 0.42^e^	31.58 ± 0.54^g^	0.21 ± 0.01^gh^
WMF + 40JKF*	22.08 ± 0.74^e^	0.35 ± 0.02^bcd^	1.65 ± 0.05^ef^	14.60 ± 0.22^cd^	48.79 ± 0.62^f^	0.25 ± 0.01^fg^
*p*	< 0.05	< 0.05	< 0.05	< 0.05	< 0.05	< 0.05

*Note:* WMF + 10JKF (90% WMF and 10% JKF), WMF + 20JKF (80% WMF and 20% JKF), WMF + 30JKF (70% WMF and 30% JKF), and WMF + 40JKF (60% WMF and 40% JKF). The values represent mean values ± standard deviation (*n* = 3). Different lowercase letters in same column indicate significant differences at 5% level.

Abbreviations: JKF, jackfruit seed powder; WMF, whole maize flour.

* Represents stiff porridge from the corresponding flour.

Calcium levels differed significantly (*p* < 0.05) among the groups, as presented in Tables 2 and 3. Sifted maize flour blended with jackfruit seed powder showed a significant (*p* < 0.05) increase in calcium content, ranging from 19.39 (for SMF + 10JKF) to 48.22 mg/100 g for SMF + 40JKF (Table 2). However, preparation of stiff porridge significantly (*p* < 0.05) reduced calcium content across all sifted maize flour blended with jackfruit seed powder. Similarly, stiff porridge prepared from WMF blended with jackfruit seed powder recorded a significant (*p* < 0.05) decrease in calcium content, ranging from 8.34 to 22.08 mg/100 g, with WMF stiff porridge at 6.23 mg/100 g.

Copper levels differed significantly (*p* < 0.05) among the groups, as presented in Tables 2 and 3. Sifted maize flour blended with jackfruit seed flour showed a significant increase in copper levels, ranging from 0.23 (for SMF + 10JKF) to 0.53 mg/100 g for SMF + 40JKF (Table 2). However, preparation of stiff porridge significantly reduced copper content across all sifted maize flour blended with jackfruit seed powder. Similarly, stiff porridge prepared from WMF blended with jackfruit seed powder recorded a significant (*p* < 0.05) decrease in copper content, ranging from 0.18 to 0.35 mg/100 g, with WMF stiff porridge at 0.08 mg/100 g.

Iron levels differed significantly (*p* < 0.05) among the groups, as presented in Tables 2 and 3. Sifted maize flour (SMF) blended with jackfruit seed powder showed a significant increase in iron content, ranging from 2.05 (for SMF + 10JKF) to 3.51 mg/100 g for SMF + 40JKF (Table 2). However, preparation of stiff porridge significantly reduced iron content across all SMF blended with jackfruit seed powder. Similarly, stiff porridge prepared from WMF blended with jackfruit seed powder recorded a significant decrease in iron content, ranging from 0.70 to 1.65 mg/100 g, with WMF stiff porridge at 0.61 mg/100 g. Jackfruit seed powder recorded the highest iron level at 5.15 mg/100 g, although the level significantly decreased to 2.09 mg/100 g after preparation of stiff porridge.

Potassium levels differed significantly (*p* < 0.05) among the groups, as presented in Tables 2 and 3. Sifted maize flour (SMF) blended with jackfruit seed powder showed a significant increase in potassium, ranging from 7.61 (for SMF + 10JKF) to 16.23 mg/100 g for SMF + 40JKF. However, preparation of stiff porridge significantly (*p* < 0.05) reduced potassium content across all SMF blended with jackfruit seed powder. Similarly, stiff porridge prepared from WMF blended with jackfruit seed powder recorded a significant (*p* < 0.05) decrease in potassium content, ranging from 7.89 to 14.60 mg/100 g, with WMF stiff porridge at 4.79 mg/100 g. Jackfruit seed powder recorded the highest potassium content at 34.91 mg/100 g, although the level significantly (*p* < 0.05) decreased to 24.92 mg/100 g after preparation of stiff porridge. The same trends were observed for magnesium and manganese content for both SMF and WMF blended with different proportions of jackfruit seed powder (Tables 2 and 3).

### 3.5 Sensory Properties of Stiff Maize Porridge Prepared From Maize Flour Fortified With Jackfruit Seed Powder

The sensory evaluation scores of stiff maize porridge prepared from sifted maize flour and whole maize flour blended with JKF were assessed using a 5‐point hedonic scale (Tables 4 and 5). Statistical analysis revealed that the blending of either sifted maize flour or whole maize flour with jackfruit seed powder significantly influenced all the sensory parameters tested (*p* ≤ 0.05). The addition of jackfruit seed powder to both sifted and whole maize flours resulted in a significant (*p* ≤ 0.05) decline in sensory parameters including aroma, appearance, taste, color, mouthfeel, texture, and overall acceptability. Furthermore, differences were not statistically significant (*p* > 0.05) across all sensory attributes between the SMF + 20JKF, SMF + 30JKF, and SMF + 40JKF samples, as well as WMF + 30JKF and WMF + 40JKF.

**TABLE 4:** Sensory evaluation scores for stiff maize porridge prepared from the sifted maize meal flour blended with different proportions of jackfruit seed powder.

Stiff porridge sample	Appearance	Color	Aroma	Mouthfeel	Taste	Texture	Overall acceptability
SMF	4.53 ± 0.67^a^	4.38 ± 0.75^a^	4.13 ± 0.75^a^	3.72 ± 0.77^a^	3.75 ± 0.92^a^	3.97 ± 1.00^a^	4.13 ± 0.66^a^
SMF + 10JKF	3.30 ± 0.94^b^	3.17 ± 0.87^b^	3.57 ± 0.93^a^	2.50 ± 0.96^b^	2.70 ± 0.97^b^	3.07 ± 1.38^b^	3.10 ± 0.70^b^
SMF + 20JKF	2.75 ± 1.02^bc^	3.00 ± 0.98^bc^	2.53 ± 0.84^b^	2.09 ± 0.93^b^	1.88 ± 0.98^c^	2.00 ± 1.02^c^	2.50 ± 0.92^c^
SMF + 30JKF	2.44 ± 0.95^c^	2.75 ± 0.95^bc^	2.72 ± 0.95^b^	2.09 ± 0.96^b^	1.88 ± 0.91^c^	2.22 ± 1.04^c^	2.28 ± 0.77^c^
SMF + 40JKF	2.18 ± 0.98^c^	2.00 ± 0.92^c^	2.54 ± 1.23^b^	1.61 ± 1.00^b^	1.54 ± 1.12^c^	2.07 ± 1.31^bc^	1.96 ± 0.93^c^
*p*	< 0.05	< 0.05	< 0.05	< 0.05	< 0.05	< 0.05	< 0.05

*Note:* Values are means ± standard deviation (*n* = 30). Means within the same column with different superscripts differ significantly (*p* < 0.05). SMF and JKF represent Sifted maize flour and Jackfruit seed powder, respectively.

**TABLE 5:** Sensory evaluation scores for stiff maize porridge prepared from the whole maize meal flour blended with different proportions of jackfruit seed powder.

Stiff porridge sample	Appearance	Color	Aroma	Mouthfeel	Taste	Texture	Overall acceptability
WMF	3.89 ± 0.85^a^	3.78 ± 0.75^a^	3.52 ± 0.94^a^	3.48 ± 0.89^a^	3.74 ± 0.90^a^	3.59 ± 1.05^a^	3.78 ± 0.70^a^
WMF + 10JKF	3.33 ± 0.88^a^	3.26 ± 0.76^a^	3.11 ± 0.85^ab^	2.56 ± 1.05^b^	2.41 ± 1.11^b^	2.41 ± 1.19^b^	2.85 ± 0.91^b^
WMF + 20JKF	2.26 ± 0.71^b^	2.52 ± 0.64^b^	2.81 ± 1.11^ab^	1.74 ± 0.76^c^	1.74 ± 0.66^c^	2.00 ± 1.00^b^	2.30 ± 0.82^bc^
WMF + 30JKF	2.22 ± 0.70^b^	2.33 ± 0.73^b^	2.89 ± 0.85^ab^	2.04 ± 0.98^bc^	1.81 ± 0.83^bc^	1.89 ± 0.93^b^	2.26 ± 0.66^c^
WMF + 40JKF	2.18 ± 0.82^b^	2.00 ± 0.86^b^	2.54 ± 1.04^b^	1.61 ± 0.69^c^	1.54 ± 0.69^c^	2.07 ± 1.02^b^	1.96 ± 0.74^c^
*p*	< 0.05	< 0.05	< 0.05	< 0.05	< 0.05	< 0.05	< 0.05

*Note:* The values are mean ± standard deviation (*n* = 30). Values in the same column with different superscript letters are significantly different (*p* < 0.05) based on Tukey's HSD test. WMF and JKF represent whole maize flour and Jackfruit seed powder, respectively.

Sifted maize flour (SMF) and whole maize flour (WMF) produced stiff maize porridge with the most preferred appearance, color, mouthfeel, texture, and taste with mean ratings above 3.40. Incorporating jackfruit seed flour introduced a grainy texture to the stiff maize porridge, which negatively influenced the mouthfeel of the stiff maize porridge from blended maize and jackfruit seed powder. The most acceptable blends were SMF + 10JKF and WMF + 10JKF. The acceptability of the blends decreased as the proportion of jackfruit seed powder increased.

## 4 Discussion

The present study provides a novel and systematic evaluation of jackfruit seed powder as a nutritious and underutilized by‐product for improving the nutritional profile of white maize‐based stiff porridge to curb malnutrition.

The findings demonstrated that blending of maize flour with jackfruit seed powder significantly increased the ash content. Despite the lower ash values in blended maize flour, as the proportions of jackfruit seed powder increased, there was an increase in total ash quantity in stiff maize porridge. According to Maskey et al. (2020), the high ash content in jackfruit seed flour (1.27%) may be attributed to its elevated mineral contents including magnesium, potassium, calcium, and trace elements such as iron in jackfruit seeds. The significant (*p* < 0.05) increase in ash level in maize‐jackfruit‐blended flours and stiff maize porridge suggests an increase in total mineral content due to fortification of maize with jackfruit seed powder. Whole maize‐jackfruit blended flours retained more ash compared to sifted maize‐jackfruit blends due to the presence of bran and germ components in whole maize flour. While enriching maize flour with jackfruit seed powder increased its mineral content, it remains important to ensure that the final formulation meets established nutritional standards to effectively support healthy growth.

Moisture content of flour is a critical quality parameter that greatly impacts the microbial stability, physical texture and sensory properties of stiff maize porridge. The results revealed that jackfruit seed powder had significantly lower moisture content and was significantly different from maize flour and maize‐jackfruit blended flour. The observed reduction in moisture with increased proportion of jackfruit seed powder is important for improving shelf life and reducing microbial susceptibility in maize‐jackfruit blended flour products (Terefe et al. 2022). Lower moisture decreases water activity, which inhibits microbial growth and enzymatic activity, thus enhancing product stability during storage. Incorporating jackfruit seed powder contributes to better moisture content, thereby improving shelf‐life of the products. However, stiff maize porridge prepared from maize‐jackfruit blended flour exhibited higher moisture content compared to its corresponding maize‐jackfruit blended flour. This increase is expected, as the cooking process involves the addition of water and gelatinization of starch, which causes the flour matrix to absorb and retain more moisture.

The addition of jackfruit seed powder to both sifted and whole maize flours increased protein content because jackfruit seeds are rich in protein content, generally ranging between 13% and 18% (Abedin et al. 2012; Maskey et al. 2020; Adan et al. 2020; Reddy et al. 2023). The protein content of stiff maize porridge prepared from maize‐jackfruit blended flours was significantly higher compared to the stiff porridge from non‐blended SMF and WMF. This is due to the utilization of protein‐rich jackfruit seed powder in fortification of SMF and WMF. Since maize has a low protein content (Kamotho et al. 2017; Chemutai et al. 2024), complementary foods emanating from maize products should be augmented by protein‐rich foods to improve their nutritional value. Proteins are vital for the prevention of protein‐energy malnutrition (PEM) frequently observed in children in developing countries. Proteins also support body growth, repair tissues, regulate metabolism through enzymes and hormones, and strengthen immune function. They also facilitate nutrient transport and contribute to cellular structure and function (Nelson and Cox 2012).

The crude fat content of maize flours (SMF and WMF) was significantly higher compared with the maize‐jackfruit blended flours and corresponding stiff porridge. In addition, the findings demonstrated a significant reduction in fat with increasing proportion of jackfruit seed powder, and this is due to the naturally low‐fat content of jackfruit seeds. Maize‐jackfruit blended flours contained significantly increased crude fat content as compared to the corresponding stiff maize porridge. Cooking of stiff maize porridge significantly reduced the fat content, and similar trends have been reported in studies showing that heat treatment and water‐based cooking can reduce fat levels in cereals and legumes due to fat loss through evaporation or absorption into cooking vessels (Eke‐Ejiofor et al. 2012). The reduction in fat content with increased ratio of JKF proportion may be beneficial for individuals managing conditions such as diabetes, obesity, or cardiovascular disease, where lower dietary fat intake is often recommended. Foods with low fat content tend to have better shelf stability, as they are less likely to become rancid during storage (Vieira et al. 2015). Fats in the diet are important in supporting overall health. They provide an energy source and facilitate the absorption of fat‐soluble vitamins such as A, D, E, and K throughout the body.

Jackfruit seed powder recorded the highest fiber content and increasing the proportion of jackfruit seed powder in composite flours for both sifted and whole maize and corresponding stiff maize porridge, resulted in a significant increase in crude fiber content. This is important because dietary fiber has many health benefits such as improved microbiota composition, metabolic parameters and synthesis beneficial metabolites, as reported in previous (Kamotho et al. 2017; Deshmukh et al. 2023). The high carbohydrate content in jackfruit seed powder and maize flour highlight their potential as reliable energy sources in the diet as reported in previous studies (Kamotho et al. 2017; Madan et al. 2022; Reddy et al. 2023). There was significant increase of carbohydrate content in SMF and WMF blended with jackfruit seed powder, whereas there was slight decrease in carbohydrate content of the stiff maize porridge, due to the reason that maize is a good source of carbohydrates and replacing maize flour with jackfruit seed powder has increased protein and reduced carbohydrate content.

An increasing trend in phenolic content was observed with higher jackfruit seed powder levels, attributed to the naturally high phenolic levels in jackfruit. Phenolic compounds are important antioxidants that play a protective role against diseases linked to oxidative stress including cardiovascular disorders, cancer, and neurodegeneration (Balasundram et al. 2006). When phenolic compounds are present in high amounts, they can lower the nutritional composition of food by hindering mineral uptake and protein digestion and may also lead to toxicity and other health issues. To mitigate these effects, various techniques like milling, soaking, fermentation, and heat treatment are commonly applied to reduce phenolic content and enhance nutrient bioavailability (Samtiya et al. 2020). In this study, phenolic content significantly decreased after cooking, suggesting that thermal processing may help mitigate the anti‐nutritional effects of phenols while retaining their beneficial properties to some extent.

Analysis of vitamin B1 content revealed that sifted and whole maize flour samples lacked detectable levels of thiamine. However, incorporation of jackfruit seed powder into sifted and whole maize flours led to a significant increase in vitamin B1. Thiamine (vitamin B1) plays a vital role in maintaining proper cellular function. It serves as a coenzyme in the breakdown of fats, carbohydrates, and proteins, playing a key role in mitochondrial energy production. Thiamine is also crucial for fatty acid oxidation, protein synthesis, and maintaining the normal function of both the central and peripheral nervous systems through its involvement in neurotransmitter production. Deficiency can cause significant health issues including Wernicke's encephalopathy, nerve damage, heart failure, and confusion, especially in malnourished individuals (Mrowicka et al. 2023). Therefore, the increase in thiamine content with jackfruit seed flour substitution suggests a potential strategy to improve the micronutrient profile of staple foods such as *Ugali*. Cooked samples exhibited a significant loss of vitamin B1 content; however, blends with higher proportions of jackfruit seed flour retained more thiamine. This confirms the sensitivity of thiamine to heat, as reported by Khamila et al. (2020) who noted that vitamins are vulnerable to degradation when subjected to heat, oxygen, or light.

The analysis of vitamin B6 content across the various maize flour formulations demonstrated a significant increase with higher jackfruit seed powder levels, yet a notable reduction was observed in the corresponding stiff maize porridge. This retention of vitamin B6 in the stiff maize porridge prepared from maize flour blended with jackfruits highlights the nutritional benefits of fortifying maize flour with jackfruit seed powder. Despite thermal losses, the stiff porridge prepared from enriched blended flours still preserved appreciable levels of B6, which is essential for amino acid metabolic processes, production of neurotransmitters, immune system function, and cognitive development. It supports red blood cell formation and helps preserve proper nerve function (Parra et al. 2018). According to Khamila et al. (2020), pyridoxine, like other vitamin B, is vulnerable to degradation when exposed to heat, light, or oxygen, explaining the reduction observed in stiff porridge.

The mineral composition of the maize–jackfruit blended flour showed a consistent improvement in mineral content with increasing proportions of jackfruit seed powder. This trend was evident across key minerals such as iron, magnesium, calcium, potassium, copper, and manganese, reflecting the naturally rich mineral profile of jackfruit seed. The analysis of stiff porridge was done on a wet weight basis and the apparent loss in mineral content following porridge preparation is attributed to dilution due to the use of water in the preparation of porridge at a ratio of 2:1 (water: flour), although some formulations retained appreciable amounts of these nutrients. These findings highlight the potential of jackfruit seed powder as a functional ingredient for combating micronutrient deficiencies, especially among communities where maize forms the dietary staple.

Considering the crucial function of iron in transporting oxygen, immune function, and cognitive development (Abbaspour et al. 2014), the observed improvements emphasize the importance of fortifying maize flour with jackfruit seed powder to help address iron deficiency in staple diets. The findings highlight that although copper content increased with increasing proportion of jackfruit seed powder, statistical differences were minimal in most formulations. Nonetheless, fortification contributed meaningfully to total copper levels, which is beneficial as an essential trace element that supports energy production, connective tissue formation, and nervous system function through its role in key cuproenzymes. It also facilitates iron metabolism and serves as an antioxidant by counteracting free radicals. Copper deficiency or imbalance may result in serious health issues, such as anemia, bone weakness, and neurological disorders (Collins 2021). Magnesium supports key bodily functions, and long‐term insufficient intake may contribute to chronic health conditions. Assessing magnesium status is challenging, as blood levels don't always reflect the body's true magnesium stores. Ensuring adequate dietary intake is vital for preventing deficiency‐related diseases (Fiorentini et al. 2021).

Sensory evaluation is essential in determining the acceptability of fortified food products as it assesses consumer perceptions of key attributes such as aroma, color, texture, taste, appearance, and overall acceptability. It typically involves numerical scoring of these specific attributes in one or more samples, with ratings commonly categorized as excellent, very good, good, fair, or poor (Kumari et al. 2015). The observed decline in sensory scores with increasing jackfruit seed powder can be attributed to several compositional and functional factors inherent in the jackfruit seed. Maskey et al. (2020) reported comparable findings, observing that the control cookies received the highest scores, which steadily declined as the jackfruit seed powder content increased. In addition to the observed changes in sensory quality, the incorporation of jackfruit seed powder may also have impacted the binding and cohesiveness of the stiff porridge matrix. Jackfruit seed powder contains starches and proteins that differ structurally from those in maize flour, potentially altering gelatinization and water absorption during cooking. These changes could result in a less cohesive or crumbly product, which may further affect texture and consumer perception. Moreover, the natural phytochemicals present in jackfruit seed powder, while nutritionally beneficial, might contribute to a bitter taste when used in higher proportions. These functional properties highlight the need for further optimization of jackfruit seed powder levels in maize‐based formulations to strike a balance between enhanced nutrition and consumer acceptability (Swami et al. 2012).

The study presented an innovative approach for food formulation by transforming low‐value jackfruit seed waste into a high‐value functional ingredient thus creating opportunities for farmers, consumers and manufacturers. Maize is a primary staple diet for hundreds of millions of people particularly across sub‐Saharan Africa. The significance of maize flour fortification in the current study lies in the ability to combat micronutrient deficiencies such as protein, minerals (iron, zinc) and vitamin B complex deficiency at a mass scale without requiring consumers to change their dietary habits. The jackfruit seeds are easily available without seasonal limitations and therefore a sustainable source of nutrients for food fortification. In addition, jackfruit seeds are considered a waste and therefore a cost‐effective strategy to enhance the nutritional value of food products. The techniques used in the pre‐processing of jackfruit seeds are easy to implement, cost‐effective and may be applicable at small‐scale community and industrial levels. There is a need to promote the adoption of jackfruit seeds in both community and industrial scales to fully harness the potential as a sustainable source of food nutrients.

## 5 Conclusion

The findings revealed that jackfruit seed powder contains significant amounts of protein, vitamins B complex (vitamin B1 and B6), and minerals compared to sifted and whole maize flours. The results demonstrated that supplementation of maize flour with jackfruit seed powder led to a significant increase in the nutritional value of stiff maize porridge. The increase in the proportion of jackfruit seed powder in maize‐jackfruit blended flours resulted in increased nutritional quality in the corresponding stiff maize porridge. The most nutrient‐rich stiff porridge was those prepared from maize flour fortified with 40% jackfruit seed powder, while the most acceptable stiff porridge based on sensory analysis was those supplemented with 10% jackfruit seed powder. The incorporation of 10% jackfruit seed powder in both SMF and WMF yielded the most acceptable stiff porridge based on texture, taste and appearance, while still offering enhanced nutritional value. These results illustrate the potential of jackfruit seed powder as a functional ingredient in the development of composite flour, offering nutritional benefits while maintaining acceptable sensory attributes. Future studies should evaluate product stability, characterize amino acid profile, micronutrient contribution, mineral bioavailability and access acceptability in broader and more relevant consumer groups.

## Author Contributions

**Felix Ochieng Odhoch:** conceptualization, methodology, investigation, validation, data curation, formal analysis, writing – original draft, writing – review and editing. **Redemtor A. Ojwang:** conceptualization, methodology, investigation, validation, supervision, funding acquisition, project administration, writing – review and editing. **Mercy W. Mwaniki:** conceptualization, methodology, investigation, validation, funding acquisition, writing – review and editing, project administration, supervision. **Betty N. Mbatia:** conceptualization, methodology, investigation, validation, funding acquisition, writing – review and editing, project administration, supervision. **Mukiri E. Gitiri:** methodology, investigation, formal analysis, writing – review and editing. **Edward K. Muge:** conceptualization, methodology, investigation, validation, funding acquisition, writing – review and editing, project administration, supervision. **Evans N. Nyaboga:** conceptualization, methodology, investigation, validation, funding acquisition, writing – review and editing, project administration, supervision.

## Funding

This work was supported by the International Foundation for Science (IFS) (Research Agreement No. I‐3‐E‐5872‐2).

## Ethics Statement

This study on stiff maize porridge fortified with jackfruit seed powder was conducted exclusively using plant materials and did not involve any human participants, human data, animals, or animal‐derived experimental procedures. Therefore, ethical approval from a human or animal ethics committee was not required for this research. All experimental procedures were carried out in accordance with relevant institutional and scientific guidelines for plant‐based studies.

## Consent

The panelists who agreed to participate in sensory evaluation of fortified stiff maize porridge signed an informed consent form before they participated in this study.

## Conflicts of Interest

The authors declare no conflicts of interest.

## Data Availability

The data that supports the finding of this study are included in this article. Further inquiries on raw data can be availed by the corresponding author upon request.

## References

[fsn372478-bib-0001] Abbaspour, N. , R. Hurrell , and R. Kelishadi . 2014. “Review on Iron and Its Importance for Human Health.” Journal of Research in Medical Sciences 19, no. 2: 164–174. PMC3999603 24778671

[fsn372478-bib-0002] Abedin, M. S. , M. M. Nuruddin , K. U. Ahmed , and A. Hossain . 2012. “Nutritive Compositions of Locally Available Jackfruit Seeds ( *Artocarpus heterophyllus* ) in Bangladesh.” International Journal of Biosciences 2, no. 8: 1–7. http://www.innspub.net.

[fsn372478-bib-0003] Adan, A. A. , R. A. Ojwang , E. K. Muge , B. K. Mwanza , and E. N. Nyaboga . 2020. “Phytochemical Composition and Essential Mineral Profile, Antioxidant and Antimicrobial Potential of Unutilized Parts of Jackfruit.” Food Research 4, no. 4: 1125–1134. 10.26656/fr.2017.4(4).326.

[fsn372478-bib-0004] Akter, F. , and M. Haque . 2019. “Jackfruit Waste: A Promising Source of Food and Feed.” Annals of Bangladesh Agriculture 23, no. 1: 91–102. 10.3329/aba.v23i1.51477.

[fsn372478-bib-0005] AOAC . 2000. “Association of Official Analytical Chemists.” In Official Methods of Analysis, 15th ed. AOAC International.

[fsn372478-bib-0006] AOAC . 2012. Official Methods of Analysis, Association of Official Analytical Chemist. 19th ed. AOAC International.

[fsn372478-bib-0007] Arefin, P. , S. Ahmed , M. S. Habib , et al. 2022. “Assessment and Comparison of Nutritional Properties of Jackfruit Seed Powder With Rice, Wheat, Barley, and Maize Flour.” Current Research in Nutrition and Food Science 10, no. 2: 544–552. 10.12944/CRNFSJ.10.2.11.

[fsn372478-bib-0008] Awata, O. , L. A. Tongoona , E. Danquah , et al. 2019. “Understanding Tropical Maize ( *Zea mays* L.): The Major Monocot in Modernization and Sustainability of Agriculture in Sub‐Saharan Africa.” International Journal of Agricultural Research 7: 32–77. 10.33500/ijaar.2019.07.004.

[fsn372478-bib-0009] Balasundram, N. , K. Sundram , and S. Samman . 2006. “Phenolic Compounds in Plants and Agri‐Industrial By‐Products: Antioxidant Activity, Occurrence, and Potential Uses.” Food Chemistry 99, no. 1: 191–203. 10.1016/j.foodchem.2005.07.042.

[fsn372478-bib-0010] Brahma, R. , and S. Ray . 2023. “Finding Out Various Potentials and Possibilities of Jackfruit Seed and Its Usage in the Industry: A Review.” Food Production, Processing and Nutrition 5, no. 1: 55. 10.1186/s43014-023-00170-z.

[fsn372478-bib-0011] Chaudhary, G. , M. Kumar , and A. R. Sehrawat . 2025. “Food Fortification: Strategy to Combat Hidden Hunger: A Systematic Review.” Food Chemistry International 1, no. 4: 384–407. 10.1002/fci2.70039.

[fsn372478-bib-0012] Chemutai, S. , M. Mburu , D. Njoroge , and V. Zettel . 2024. “Effects of Ugali Maize Flour Fortification With Chia Seeds ( *Salvia hispanica* L.) on Its Physico‐Chemical Properties and Consumer Acceptability.” Foods 13, no. 4: 543. 10.3390/foods13040543. PMC10888471 38397520

[fsn372478-bib-0013] Collins, J. F. 2021. “Copper Nutrition and Biochemistry and Human (Patho)physiology.” Advances in Food and Nutrition Research 96: 311–364. 10.1016/bs.afnr.2021.01.005. 34112357

[fsn372478-bib-0014] Deshmukh, R. , A. Nadaf , W. A. Ansari , K. Singh , and H. Sonah , eds. 2023. Biofortification in Cereals: Progress and Prospects. Springer Singapore. 10.1007/978-981-19-4308-9.

[fsn372478-bib-0043] Eke‐Ejiofor, J. , D. B. Kiin–Kabari , and E. C. Chukwu . 2012. “Effect of Processing Method on the Proximate, Mineral and Fungi Properties of Groundnut (*Arachis hypogea*) Seed.” Journal of Agriculture and Biological Sciences 3, no. 1: 257–261.

[fsn372478-bib-0015] Ekpa, O. , N. Palacios‐Rojas , G. Kruseman , V. Fogliano , and A. R. Linnemann . 2019. “Sub‐Saharan African Maize‐Based Foods ‐ Processing Practices, Challenges and Opportunities.” Food Reviews International 35, no. 7: 609–639. 10.1080/87559129.2019.1588290.

[fsn372478-bib-0016] Fiorentini, D. , C. Cappadone , G. Farruggia , and C. Prata . 2021. “Magnesium: Biochemistry, Nutrition, Detection, and Social Impact of Diseases Linked to Its Deficiency.” Nutrients 13, no. 4: 1136. 10.3390/nu13041136. PMC8065437 33808247

[fsn372478-bib-0045] Kabubii, Z. N. , J. M. Mbaria , M. P. Mathiu , J. M. Wanjohi , and E. N. Nyaboga . 2023. “Evaluation of Seasonal Variation, Effect of Extraction Solvent on Phytochemicals and Antioxidant Activity on *Rosmarinus officinalis* Grown in Different Agro‐Ecological Zones of Kiambu County, Kenya.” CABI Agriculture and Bioscience 4, no. 1. 10.1186/s43170-023-00141-x.

[fsn372478-bib-0017] Kamotho, S. N. , F. M. Kyallo , and D. N. Sila . 2017. “Fortification of Maize Flour With Grain Amaranth for Improved Nutrition.” African Journal of Food, Agriculture, Nutrition and Development 17, no. 4: 12574–12588. 10.18697/ajfand.80.15945.

[fsn372478-bib-0018] Kemigisha, P. 2025. “Navigating the Gender Dynamics of Food and Nutrition Security in Southwestern Uganda.” Academia Nutrition and Dietetics 2, no. 2. 10.20935/AcadNutr7645.

[fsn372478-bib-0044] Khamila, S. , D. N. Sila , and A. Makokha . 2020. “Compliance Status and Stability of Vitamins and Minerals in Fortified Maize Flour in Kenya.” Scientific African 8: e00384. 10.1016/j.sciaf.2020.e00384.

[fsn372478-bib-0020] Kumari, V. , S. Divakar , M. Ukkruu , and P. V. Nandini . 2015. “Development of Raw Jackfruit Based Noodles.” Food Science Research Journal 6, no. 2: 326–332.

[fsn372478-bib-0021] Madan, S. G. , A. K. Singh , R. N. Shukla , H. Masih , and S. Tewari . 2022. “Physico‐Chemical Properties of Different Corn ( *Zea mays* ) Verities.” International Journal of Food and Nutritional Sciences 11, no. 3: 557–564.

[fsn372478-bib-0022] Maskey, B. , S. Subedi , and N. Shrestha . 2020. “Effect of Incorporation of Jackfruit ( *Artocarpus heterophyllus* ) Seed Flour on the Quality of Cookies.” Dristikon: A Multidisciplinary Journal 10, no. 1: 60–72.

[fsn372478-bib-0024] Mohamad, S. F. , F. Mohd Said , M. S. Abdul Munaim , S. Mohamad , and W. M. A. WanSulaiman . 2019. “Proximate Composition, Minerals Contents, Functional Properties of Mastura Variety Jackfruit ( *Artocarpus heterophyllus* ) Seeds and Lethal Effects of Its Crude Extract on Zebrafish ( *Danio rerio* ) Embryos.” Food Research 3, no. 5: 546–555.

[fsn372478-bib-0025] Mrowicka, M. , J. Mrowicki , G. Dragan , and I. Majsterek . 2023. “The Importance of Thiamine (Vitamin B1) in Humans.” Bioscience Reports 43, no. 10: BSR20230374. 10.1042/BSR20230374. PMC10568373 37389565

[fsn372478-bib-0026] Murage, M. W. , E. K. Muge , B. N. Mbatia , and M. W. Mwaniki . 2021. “Development and Sensory Evaluation of Omega‐3‐Rich Nile Perch Fish Oil‐Fortified Yogurt.” International Journal of Food Science 2021: 1–7. 10.1155/2021/8838043. PMC7899769 33628772

[fsn372478-bib-0028] Nelson, D. L. , and M. M. Cox . 2012. Lehninger Principles of Biochemistry. 6th ed. W. H. Freeman and Company. bfwpub.com.

[fsn372478-bib-0029] Noor, F. , M. J. Rahman , M. S. Mahomud , M. S. Akter , M. A. I. Talukder , and M. Ahmed . 2014. “Physicochemical Properties of Flour and Extraction of Starch From Jackfruit Seed.” International Journal of Nutrition and Food Sciences 3, no. 4: 347–354. 10.11648/j.ijnfs.20140304.27.

[fsn372478-bib-0030] Parra, M. , S. Stahl , and H. Hellmann . 2018. “Vitamin B6 and Its Role in Cell Metabolism and Physiology.” Cells 7, no. 7: 84. 10.3390/cells7070084. PMC6071262 30037155

[fsn372478-bib-0031] Phan‐Thien, K. Y. , G. C. Wright , and N. A. Lee . 2012. “Inductively Coupled Plasma‐Mass Spectrometry (ICP‐MS) and ‐Optical Emission Spectroscopy (ICP‐OES) for Determination of Essential Minerals in Closed Acid Digestates of Peanuts ( *Arachis hypogaea* L.).” Food Chemistry 134, no. 1: 453–460. 10.1016/j.foodchem.2012.02.095.

[fsn372478-bib-0032] Prasanna, B. M. , S. K. Vasal , K. Bisetegn , and N. N. Singh . 2001. “Quality Protein Maize.” Current Science 81, no. 10: 1308–1319.

[fsn372478-bib-0033] Ranasinghe, R. A. S. N. , S. D. T. Maduwanthi , and R. A. U. J. Marapana . 2019. “Nutritional and Health Benefits of Jackfruit ( *Artocarpus heterophyllus* Lam.): A Review.” International Journal of Food Science 2019: 4327183. 10.1155/2019/4327183. PMC6339770 30723733

[fsn372478-bib-0034] Reddy, K. , G. P. Sujatha , and R. Narayanan . 2023. “Effect of Various Processing Methods on Jack Fruit ( *Artocarpus heterophyllus* ) Seed Flours.” Biological Forum—An International Journal 15, no. 11: 168–173.

[fsn372478-bib-0035] Samtiya, M. , R. E. Aluko , and T. Dhewa . 2020. “Plant Food Anti‐Nutritional Factors and Their Reduction Strategies: An Overview.” Food Production, Processing and Nutrition 2, no. 1: 6. 10.1186/s43014-020-0020-5.

[fsn372478-bib-0036] Sarwar, S. , F. T. Zohora , O. Khatun , et al. 2020. “Estimation of B‐Vitamins (B1, B2, B3 and B6) by HPLC in Vegetables Including Ethnic Selected Varieties of Bangladesh.” Pharmacy & Pharmacology International Journal 8: 16–23. 10.15406/ppij.2020.08.00275.

[fsn372478-bib-0038] Swami, S. B. , N. J. Thakor , P. M. Haldankar , and S. B. Kalse . 2012. “Jackfruit and Its Many Functional Components as Related to Human Health: A Review.” Comprehensive Reviews in Food Science and Food Safety 11, no. 6: 565–576. 10.1111/j.1541-4337.2012.00210.x. 33467822

[fsn372478-bib-0039] Terefe, Z. K. , M. Omwamba , and J. Nduko . 2022. “Optimization of Extrusion Cooking Variables for Production of Protein Enriched Maize‐Cassava Leaf Composite Instant Porridge Flour.” International Journal of Food Science and Agriculture 6, no. 1: 93–106.

[fsn372478-bib-0040] Vieira, S. A. , D. J. McClements , and E. A. Decker . 2015. “Challenges of Utilizing Healthy Fats in Foods.” Advances in Nutrition 6, no. 3: 309S–317S. 10.3945/an.114.006965. PMC4424769 25979504

[fsn372478-bib-0041] Yilmaz, H. , and A. Yilmaz . 2025. “Hidden Hunger in the Age of Abundance: The Nutritional Pitfalls of Modern Staple Crops.” Food Science & Nutrition 13, no. 2: e4610. 10.1002/fsn3.4610. PMC11788495 39901987

[fsn372478-bib-0042] Zaria, C. , V. Aina , B. Sambo , et al. 2012. “Determination of Nutritional and Anti‐Nutrient Content of *Vitis vinifera* (Grapes) Grown in Bomo Area.” Advance Journal of Food Science and Technology 4, no. 6: 445–448.

